# Supplementary material for: Cost-effectiveness of the Healthy Options group psychosocial intervention for perinatal women living with HIV and depression in Tanzania
Source: PLOS Ment Health. 2024 Dec 2;1(7):e0000066. doi: 10.1371/journal.pmen.0000066 (PMC12798287; doi:10.1371/journal.pmen.0000066)
Supplement: S1 Table — (DOCX) [file pmen.0000066.s001.docx]

**S1 Table: Resource inputs and costs (in US$)**

| **Inputs** | **Details** | **Unit Cost (US$)** |
| --- | --- | --- |
| **Buildings/Space** | Assumed 64sq.m per facility | 305.3 |
| **Furniture** | 3 chairs for facilitators per facility | 17.4 |
|  | 1 wooden table per facility | 65.4 |
|  | 4 wooden benches for clients per facility | 32.7 |
| **Human resources** | 22 Lay facilitators | 6,166.71 each for 2 years/ 256.95 per month |
|  | 5 Supervisors | 9,336.15 each for 2 years/ 389.01 per month |
|  | 1 Clinical psychiatrist | 3,523.45 |
|  | 1 Study coordinator | 1,921.97 per month |
| **Office consumables** | Office supplies, 2 laptops, 1 UPS | 3,911.99 |
| **Training (peer facilitators, supervisors and health care providers)** |  |  |
| Printing | Materials printed include; each site two standardized manual for screening, diagnosis and management of depression | 306.44 |
| Transport | Reimbursement of travel cost to peer facilitators, supervisors and health care providers | 17.4 per day |
| Stipend | Time compensation for facilitators, supervisors and health care providers | Included with transport (above) |
| **Therapy sessions** |  |  |
| Travel reimbursement | Travel reimbursement (participants) | 5.5 per day |
| Refreshments | Refreshments during group sessions | 8,032.34 |
| **Communication** | Phone and internet | 5,125.93 |
