# Supplementary material for: Cost-effectiveness of the Healthy Options group psychosocial intervention for perinatal women living with HIV and depression in Tanzania
Source: PLOS Ment Health. 2024 Dec 2;1(7):e0000066. doi: 10.1371/journal.pmen.0000066 (PMC12798287; doi:10.1371/journal.pmen.0000066)
Supplement: Attachment — Submitted filename: Response to Reviewers.docx [file pmen.0000066.s002.docx]

**UNITED REPUBLIC OF TANZANIA**

MINISTRY OF EDUCATION, SCIENCE AND TECHNOLOGY

MUHIMBILI UNIVERSITY OF HEALTH AND ALLIED SCIENCES

**SCHOOL OF PUBLIC HEALTH AND SOCIAL SCIENCES**

**DEPARTMENT OF BEHAVIOURAL SCIENCES**

12^th^ September 2024

**Editor-in-Chief**

**PLOS Mental Health**

Dear Editor,

Thank you very much for the opportunity to revise our manuscript. I am pleased to resubmit a research article titled “*Cost-effectiveness of the Healthy Options group psychosocial intervention for perinatal women living with HIV and depression in Tanzania*” for publication consideration in PLOS Mental Health after working through editor’s and reviewer’s comments. We appreciate each point raised by the reviewers; we have provided a point-by-point response highlighted in blue font.

**Comment 1:** Journal Requirements:
1. We note that your Data Availability Statement is currently as follows: The dataset supporting the conclusions of this article is included within the article.

Please confirm at this time whether or not your submission contains all raw data required to replicate the results of your study. Authors must share the “minimal data set” for their submission. PLOS defines the minimal data set to consist of the data required to replicate all study findings reported in the article, as well as related metadata and methods (https://journals.plos.org/plosone/s/data-availability#loc-minimal-data-set-definition).
 
 For example, authors should submit the following data:

 - The values behind the means, standard deviations and other measures reported;
 - The values used to build graphs;

 - The points extracted from images for analysis.
 
Authors do not need to submit their entire data set if only a portion of the data was used in the reported study.
 
If your submission does not contain these data, please either upload them as Supporting Information files or deposit them to a stable, public repository and provide us with the relevant URLs, DOIs, or accession numbers. For a list of recommended repositories, please see https://journals.plos.org/plosone/s/recommended-repositories.
 
If there are ethical or legal restrictions on sharing a de-identified data set, please explain them in detail (e.g., data contain potentially sensitive information, data are owned by a third-party organization, etc.) and who has imposed them (e.g., an ethics committee). Please also provide contact information for a data access committee, ethics committee, or other institutional body to which data requests may be sent. If data are owned by a third party, please indicate how others may request data access.

**Response: We have added missing data on Supplemental table 1 (previously Table 1: Resource inputs and unit costs (in US$)) as suggested by reviewer number 2, by indicating the amounts in all input categories instead of labelling as "lump sum". We now hope the data presented in tables, details contained in manuscript text (including assumptions), and supplemental file allow for replication of the results of our study.**

Additional Editor Comments (if provided): Kindly address the comments raised by reviewers.

[Note: HTML markup is below. Please do not edit.]

Reviewers' comments:

Reviewer's Responses to Questions

**Comments to the Author**

1. Does this manuscript meet PLOS Mental Health’s [publication criteria](https://journals.plos.org/mentalhealth/s/criteria-for-publication)? Is the manuscript technically sound, and do the data support the conclusions? The manuscript must describe methodologically and ethically rigorous research with conclusions that are appropriately drawn based on the data presented.

Reviewer #1: Yes

Reviewer #2: Yes

**Response:** **We appreciate the positive feedback.**

 2. Has the statistical analysis been performed appropriately and rigorously?

Reviewer #1: Yes

Reviewer #2: Yes

**Response:** **We appreciate the positive feedback.**

 3. Have the authors made all data underlying the findings in their manuscript fully available (please refer to the Data Availability Statement at the start of the manuscript PDF file)?

The [PLOS Data policy](https://journals.plos.org/mentalhealth/s/data-availability) requires authors to make all data underlying the findings described in their manuscript fully available without restriction, with rare exception. The data should be provided as part of the manuscript or its supporting information, or deposited to a public repository. For example, in addition to summary statistics, the data points behind means, medians and variance measures should be available. If there are restrictions on publicly sharing data—e.g. participant privacy or use of data from a third party—those must be specified.

Reviewer #1: Yes

Reviewer #2: No

**Response: As in our response to journal requirement above, we have added missing data on Supplemental table 1 (previously Table 1: Resource inputs and unit costs (in US$)) as suggested by reviewer number 2, by indicating the amounts in all input categories instead of labelling as "lump sum". We now hope the data presented in tables, details contained in manuscript text (including assumptions), and supplemental file allow for replication of the results of our study.**

4. Is the manuscript presented in an intelligible fashion and written in standard English?

PLOS Mental Health does not copyedit accepted manuscripts, so the language in submitted articles must be clear, correct, and unambiguous. Any typographical or grammatical errors should be corrected at revision, so please note any specific errors here.

Reviewer #1: Yes

Reviewer #2: Yes

**Response:** **We appreciate the positive feedback.**

5. Review Comments to the Author

Please use the space provided to explain your answers to the questions above. You may also include additional comments for the author, including concerns about dual publication, research ethics, or publication ethics. (Please upload your review as an attachment if it exceeds 20,000 characters)

**Reviewer #1: RE: PMEN-D-24-00182**
Cost-effectiveness of the Healthy Options group psychosocial intervention for perinatal women living with HIV and depression in Tanzania

SUMMARY: This paper reported the clinical benefit and economic assessment of the Healthy Options (intervention title) intervention from a cluster randomized clinical trial to address mental health issues for perinatal women living with HIV in Tanzania. All participants received enhanced usual care for depression treatment. Intervention group received, in extra, problem-solving therapy to promote awareness of one’s own strengths and assertive communication skills, acknowledging problems and identifying strategies for addressing them, focusing on positive thinking approaches and using dreaming to develop action plans and foster hope. Cognitive behavioral therapy was additionally provided to only those with elevated depression in the intervention group. The trial showed an improvement in the clinical outcomes—survival and depression. Standard methods were used and reproducible. Among those, the usage of PHQ-9 as a common depression measure helps for future literature synthesis for multiple purposes. It is much appreciated to have a “negative cost-effectiveness” finding reported since the economic evaluation literature has had an inclination on reporting “positive cost-effectiveness” finding, aka a publication bias. Healthy Options was not cost-effective compared to usual care under the standard willingness to pay threshold of up to 3 times of the country’s GDP per capita.

**Response:** **We appreciate the positive feedback.**

MAJOR ISSUES
• Main goal of the paper: a) As the title holds on cost-effectiveness, much of report on study outcomes are deemed redundant. Should only study outcomes on death and depression be reported because these are key drivers to the estimate of the denominator of the incremental cost-effectiveness ration. b) The paper is informational though. If no other paper reports all study outcomes, this paper may use an alternate title to include “Clinical and economic benefits of Healthy Options”...

**Response: We have taken option (a) into account and made changes to the manuscript report on study outcomes. We now only report study outcomes on death and depression as these are the key drivers to the estimate of the denominator of the incremental cost-effectiveness ratio. Another paper (**[***A peer-facilitated psychological group intervention for perinatal women living with HIV and depression in Tanzania-Healthy Options: A cluster-randomized controlled trial***](https://www.ncbi.nlm.nih.gov/pmc/articles/PMC9746973/)**) has reported the other study outcomes.**

• There are too many tables. Consider deleting redundant and combining them. Table 1 can be in Supplemental. Table 2 can be included in the base-case analysis reporting for the cost-effectiveness. Table 3. Delete. Table 5: Delete. Table 7 and 8 may be combined (Also, I don’t think there was a need to report unadjusted costs. I would suggest proceeding with the reporting with the adjusted number and holding as is the methods discussing the unadjusted and adjusted costs).

**Response: We have made Table 1 supplemental (now Supplemental table 1). We have combined Table 2 with the base-case analysis reporting for the cost-effectiveness. We have deleted Table 3 and Table 5. We have combined Table 7 and 8. Therefore, we now have Table 1 reporting DALYs for control and intervention, Table 2 reporting inflation adjusted cost, Table 3 reporting base-case cost-effectiveness analysis of Healthy Options intervention, and Table 4 reporting sensitivity analysis.**

**We have removed reporting of unadjusted costs; we report adjusted numbers and hold as is the methods discussing the unadjusted and adjusted costs.**

• Table 9: Please, always use “incremental” cost-effectiveness ratio. Economic evaluation is an incremental analysis. Better wording in the first column is needed if readers would like to skim through the tables first. Cost -> Implementation cost or Costs of Healthy Options (implementation). It is confusing to use a negative DALY in this table. Should this DALY number be positive since the intervention group had better survival and lower depression scores?

**Response: We have improved wording on the first column to read Incremental Cost of HO (US$), Incremental Effectiveness of HO (DALYs averted), and Incremental Cost-Effectiveness Ratio of HO (US$). We have also removed the negative value since the intervention group had better survival and lower depression scores hence averted more DALYs.**

• The authors separated intervention vs. non-intervention costs. However, how could you differentiate them? Resources used were recorded; but how did the recording help you arrive at the estimators such as 75%?

**Response:** **At the time of the intervention, both consumables and communication were shared with other non-intervention activities such as research ethical clearance, randomization and other research-related activities, therefore we hypothesized the amount of non-intervention cost for these items would be 25%. It was a decision made by the team based on experience of how these resources (consumables and communication) were utilized at the time.**

MINOR ISSUES
• Line 392: You can quickly and roughly convert £1200 into USD2017 for readers’ ease

**Response:** **We have looked at historical data from various online sources and converted the value to approximately $1540, adopting an exchange rate of £1=USD1.28 for 2017. This has been added to the discussion.**

• Use consistent wording across tables

**Response:** **We have cross-checked tables for wording consistency and made some edits.**

**Reviewer #2:** In consideration of no.3 (above) and Table 1 of the manuscript please indicate the amount instead of labeling it as "lump sum" for the sake of clarity and replication. Otherwise the manuscript is well written.

**Response:** **For the sake of clarity and replication, we have added missing data on Supplemental table 1 (previously Table 1: Resource inputs and unit costs (in US$)) as suggested, by indicating the amounts in all input categories instead of labelling as "lump sum". We now hope the data presented in tables, details contained in manuscript text (including assumptions), and supplemental file allow for replication of the results of our study.**

6. PLOS authors have the option to publish the peer review history of their article ([what does this mean?](https://journals.plos.org/mentalthealth/s/editorial-and-peer-review-process#loc-peer-review-history)). If published, this will include your full peer review and any attached files.

**Do you want your identity to be public for this peer review?** If you choose “no”, your identity will remain anonymous but your review may still be made public.


For information about this choice, including consent withdrawal, please see our [Privacy Policy](https://www.plos.org/privacy-policy).

Reviewer #1: No

Reviewer #2: **Yes:** Mah Wasi Asombang

[NOTE: If reviewer comments were submitted as an attachment file, they will be attached to this email and accessible via the submission site. Please log into your account, locate the manuscript record, and check for the action link "View Attachments". If this link does not appear, there are no attachment files.]

**Response: We checked the action link, there was no attachment.**

While revising your submission, please upload your figure files to the Preflight Analysis and Conversion Engine (PACE) digital diagnostic tool, https://pacev2.apexcovantage.com/. PACE helps ensure that figures meet PLOS requirements. To use PACE, you must first register as a user. Registration is free. Then, login and navigate to the UPLOAD tab, where you will find detailed instructions on how to use the tool. If you encounter any issues or have any questions when using PACE, please email PLOS at figures@plos.org. Please note that Supporting Information files do not need this step.

**Response: Our manuscript does not contain any figure files.**

We hope our revisions sufficiently address all comments. We look forward to positive feedback from you.

Sincerely,

**Happiness P. Saronga**

**Corresponding Author**
