# Supplementary material for: Cost-effectiveness of the Healthy Options group psychosocial intervention for perinatal women living with HIV and depression in Tanzania
Source: PLOS Ment Health. 2024 Dec 2;1(7):e0000066. doi: 10.1371/journal.pmen.0000066 (PMC12798287; doi:10.1371/journal.pmen.0000066)
Supplement: Attachment — Submitted filename: Response to Reviewers.docx [file pmen.0000066.s003.docx]

**UNITED REPUBLIC OF TANZANIA**

MINISTRY OF EDUCATION, SCIENCE AND TECHNOLOGY

MUHIMBILI UNIVERSITY OF HEALTH AND ALLIED SCIENCES

**SCHOOL OF PUBLIC HEALTH AND SOCIAL SCIENCES**

**DEPARTMENT OF BEHAVIOURAL SCIENCES**

16^th^ October 2024

**Editor-in-Chief**

**PLOS Mental Health**

Dear Editor,

Thank you very much for the opportunity to revise our manuscript. I am pleased to resubmit a research article titled “*Cost-effectiveness of the Healthy Options group psychosocial intervention for perinatal women living with HIV and depression in Tanzania*” for publication consideration in PLOS Mental Health after working through editor’s comments. We have provided a point-by-point response highlighted in blue font.

Journal Requirements:

1. Please review your reference list to ensure that it is complete and correct. If you have cited papers that have been retracted, please include the rationale for doing so in the manuscript text, or remove these references and replace them with relevant current references. Any changes to the reference list should be mentioned in the rebuttal letter that accompanies your revised manuscript. If you need to cite a retracted article, indicate the article’s retracted status in the References list and also include a citation and full reference for the retraction notice.

**Response: We have reviewed the reference list and made sure all citations are complete and correct. We have added references number 34, 35 and 36 to support our discussion.**

Additional Editor Comments (if provided):

The responses to reviewers are strong and this paper is ready for publication. On a minor copy-edit level, may I kindly suggest you spell out the intervention name rather than use the acronym "HO" throughout?

**Response: We have spelled out the intervention name as suggested, changes have been made throughout the manuscript.**


[Note: HTML markup is below. Please do not edit.]

Reviewers' comments:


[NOTE: If reviewer comments were submitted as an attachment file, they will be attached to this email and accessible via the submission site. Please log into your account, locate the manuscript record, and check for the action link "View Attachments". If this link does not appear, there are no attachment files.]

**Response: There are no attachments on the submission site.**

While revising your submission, please upload your figure files to the Preflight Analysis and Conversion Engine (PACE) digital diagnostic tool, https://pacev2.apexcovantage.com/. PACE helps ensure that figures meet PLOS requirements. To use PACE, you must first register as a user. Registration is free. Then, login and navigate to the UPLOAD tab, where you will find detailed instructions on how to use the tool. If you encounter any issues or have any questions when using PACE, please email PLOS at figures@plos.org. Please note that Supporting Information files do not need this step.

**Response: Our manuscript does not contain any figure files.**

We hope our revisions sufficiently address all comments. We look forward to positive feedback from you.

Sincerely,

**Happiness P. Saronga**

**Corresponding Author**
